# Are physiotherapists employing person-centred care for people with dementia? An exploratory qualitative study examining the experiences of people with dementia and their carers

**DOI:** 10.1186/s12877-018-0756-9

**Published:** 2018-03-02

**Authors:** Abigail J. Hall, Lisa Burrows, Iain A. Lang, Ruth Endacott, Victoria A. Goodwin

**Affiliations:** 1 NIHR CLAHRC South West Peninsula, University of Exeter Medical School, Exeter, UK; 2 NIHR CLAHRC South West Peninsula, Plymouth University, Plymouth, UK; 3 School of Nursing and Midwifery (Faculty of Health & Human Sciences), Plymouth University, Plymouth, UK

**Keywords:** Physiotherapy, Dementia, Pcc, Patient centred care, Satisfaction, Carer

## Abstract

**Background:**

People with dementia may receive physiotherapy for a variety of reasons. This may be for musculoskeletal conditions or as a result of falls, fractures or mobility difficulties. While previous studies have sought to determine the effectiveness of physiotherapy interventions for people with dementia, little research has focused on the experiences of people receiving such treatment. The aim of this study was to gain an in-depth understanding of people’s experiences of receiving physiotherapy and to explore these experiences in the context of principles of person-centred care.

**Methods:**

Semi-structured interviews were undertaken with people with dementia or their carers between September 2016 and January 2017. A purposive sampling strategy recruited participants with dementia from the South West of England who had recently received physiotherapy. We also recruited carers to explore their involvement in the intervention. Thematic analysis was used to analyse the data.

**Results:**

A total of eleven participants were recruited to the study. Six people with dementia were interviewed and five interviews undertaken separately with carers of people with dementia. Three themes were identified. The first explores the factors that enable exercises to be undertaken successfully, the second deals with perceived resource pressures, and the final theme “the physiotherapy just vanished” explores the feeling of abandonment felt when goals and expectations of physiotherapy were not discussed. When mapped against the principles of person-centred care, our participants did not describe physiotherapy adopting such an approach.

**Conclusion:**

Lack of a person-centred care approach was evident by ineffective communication, thus failing to develop a shared understanding of the role and aims of physiotherapy. The incorporation of person-centred care may help reduce the frustration and feelings of dissatisfaction that some of our participants reported.

**Electronic supplementary material:**

The online version of this article (10.1186/s12877-018-0756-9) contains supplementary material, which is available to authorized users.

## Background

Warnings of a global dementia epidemic and its consequences have grown in recent years. A significant increase in people diagnosed with dementia is being reported in the literature, with figures suggesting the incidence doubling every twenty years to reach over 130 million people living with dementia by 2050 [1]. However, despite an apparent recent increase in interest in dementia, fears of an epidemic are not new. Concerns about a “silent epidemic” were first reported in the 1980s [2] and despite highlighting the need for more research addressing the causes, pathogenesis and medical interventions, there was a reported lack of consideration of the importance of non-medical interventions. This pattern continued over the ensuing decades, with the majority of research still focusing on medical interventions to delay onset of dementia or reduce the associated symptoms [3]. More recently, there has been an increasing growth of research looking at non-medical interventions to improve management of people with dementia, indeed a significant volume of research over the last decade has demonstrated that psychosocial interventions can be as effective as pharmacological therapies [4].

Historically, research in dementia either largely neglected subjective experiences or was comprised of carers’ opinions [5], further damaging the belief that involving people with dementia in research is feasible. A review commissioned by the Australian Government highlighted the need for a greater understanding of the experiences and needs of people with dementia and their carers [6] and was echoed by the recommendations of the James Lind Alliance dementia research priority setting review [7].

The introduction of person-centered care (PCC) has resulted in growing importance that people with dementia’s experiences should be explored through research [8] as well as the incorporation of patient’s opinions in clinical practice. It has been suggested as a way of improving outcomes for people with dementia [9] and was a concept first introduced by Kitwood [10], now reflected in a well-established biopsychosocial approach to diagnosis and management of the care of older people in the UK [11]. While a clear definition of PCC is not established, the principles suggest that patients are people and should not be classified or treated according to their disease alone, but their subjective experiences in relation to their environment, situation and future plans should be considered [12]. In 2006, the National Institute for Health and Clinical Excellence instructed acute hospital Trusts in the UK to provide services that were aligned with the principles of PCC [13] and was further supported by the National Dementia Strategy [14] which sought to ensure that significant improvements were made to dementia services. These encompassed three key aspects including improved awareness, earlier diagnosis and intervention, and a higher quality of care [14].

Gait impediments, reduced balance and impaired postural control [15] in combination with impairments in cognition lead to greater risk of falls and fractures for people with dementia [16], increasing the likelihood of a person with dementia requiring physiotherapy. Despite the suggested importance of incorporating PCC into treatment plans in order to provide effective treatment, the extent to which PCC is experienced by patients receiving physiotherapy is not clear. A previous study undertaken by the authors suggested that physiotherapists were trying to incorporate PCC into the management of people with dementia, but were often limited by lack of knowledge or resource limitations [17].

In light of the lack of research in this area, the aim of this study was to explore the experiences of people with dementia, and their carers, of the physiotherapy they received as part of a rehabilitation program. Furthermore, it aimed to explore what factors were important to people with dementia and their carers in relation to physiotherapy in order improve adherence and ability to engage in physiotherapy, while also considering whether the principles of PCC are experienced by those receiving it. For the purpose of this study, we will use the term “carer” to include relatives, next of kin or friends of the person with dementia.

## Methods

An inductive qualitative approach was used as it enabled in depth exploration of participants’ experiences and perspectives, with the aim to develop new theory, due to an absence of existing research. To gain an in-depth understanding of the experiences of people receiving physiotherapy we undertook semi-structured interviews with people with dementia in the South West of England between September 2016 and January 2017. We also interviewed carers of people with dementia to explore their experiences of being involved in the process of physiotherapy and used thematic analysis to make sense of the data.

## Recruitment and participants

**Fig. 1:**
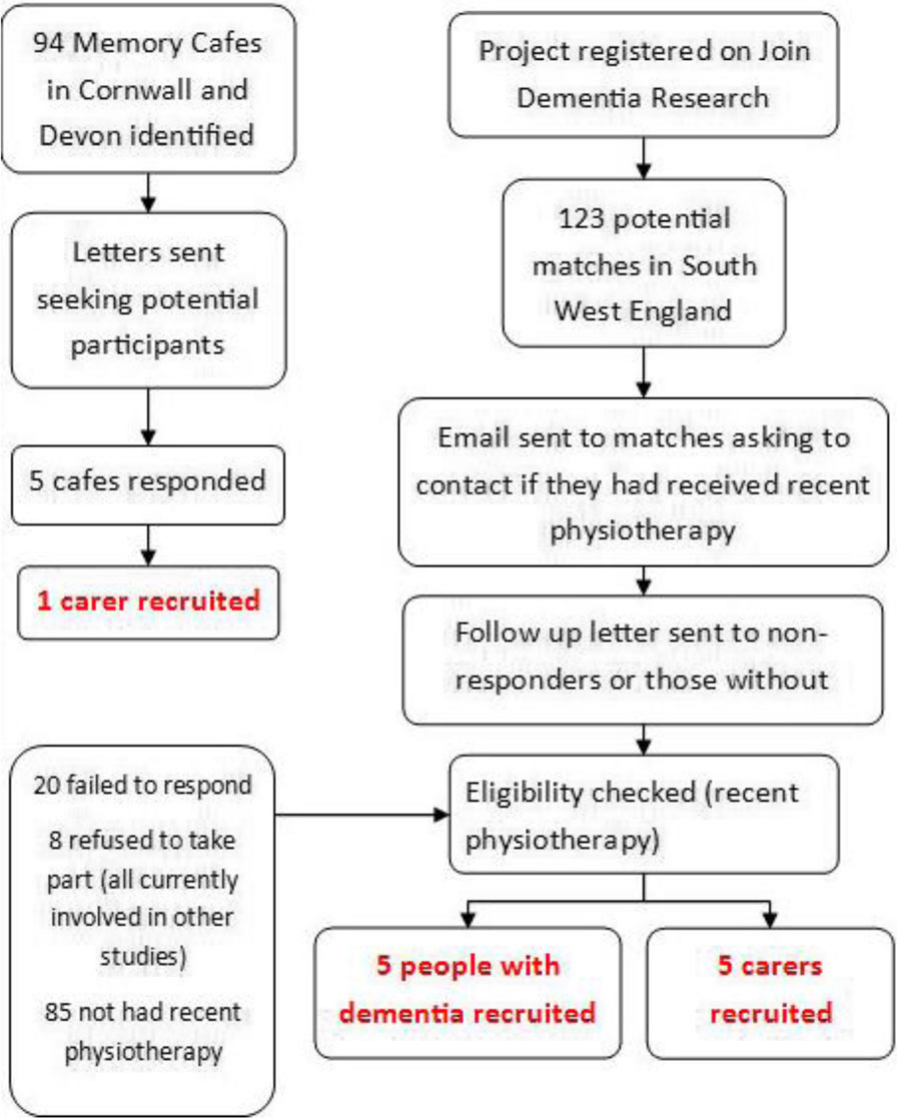
Recruitment flowchart

A purposive sampling strategy was employed, with participant inclusion based on their experiences. We included participants who had received physiotherapy within the last six months, had a diagnosis of dementia and were able to give informed consent to participate in the study. We also recruited carers of people with dementia who had experience of their relative having received physiotherapy. Recruitment utilised two strategies. Firstly, letters were sent to Memory Cafés in Devon and Cornwall. In addition, the project was registered on the “Join Dementia Research” website (JDR) (www.joindementiaresearch.nihr.ac.uk) where people with dementia can register their interest in being involved in research projects. All people registered who lived in the South West of England were sent an email to determine their potential involvement in the project. A letter was sent to those who did not have an email address or as a follow-up to those who did not respond to the initial email. The potential participants were asked to contact the research team should they have received physiotherapy recently and were happy to discuss their experiences (see Fig. 1). Recruitment and interviewing continued until no new data was emerging. It is suggested that when no new data is emerging, there will be no new coding strategies and therefore no new themes will be generated [18]. However, it is suggested that the number of emergent themes are potentially limitless [19] due to the unique interpretations and meanings that people make of social situations, so there are potentially always new interpretations [20]. A pragmatic approach suggests that an adequate sample size is one that sufficiently answers the research question [21] and places less emphasis on the number of samples required, therefore sampling ceased when sufficient data had been generated to answer the research question.

Eleven participants were recruited to the study, of whom six were people with dementia (two of these interviews were in conjunction with their carer) and five were carers of people with dementia (see Table 1). The severity of dementia was not formally recorded but all participants were able to understand the purpose of the research and able to provide written informed consent, which would suggest that they had mild to moderate dementia, although classification according to the severity of dementia was deemed unnecessary and inappropriate given the study aims.

**Table 1:** Characteristics of person receiving physiotherapy

		Number of participants
Location of physiotherapy (NB several participants received input in more than one setting)	In-patient	7
	Out-patient	3
	Community	7
Type of dementia	Alzheimer’s	7
	Lewy Body	1
	Vascular	1
	Mixed	1
	Unsure	1
Residential status	Home – alone	1
	Home – partner	7
	Nursing home	2
	Residential home	1
Gender of person with dementia	Male	3
	Female	8
Age of person with dementia	65–70	2
	71–80	5
	81–90	3
	90+	1
Type of physiotherapy	Private	1
	National Health Service	6
	(NHS)	
	Both	4

## Data collection and analysis

Interviews were undertaken with participants in a place and at a time of their choosing, usually their own home, and employed open-ended questions, which were broad in nature in order to elicit rich responses from participants. A relative or a carer could be present if the person chose. A topic guide was used to structure the interview (see Additional file 1) so although the participants were asked the same initial questions, they were phrased in a way that would encourage participants to describe their experiences in their own words. The interviewer (AH) is an experienced physiotherapist with significant experience working with people with dementia, none of the participants were known to the interviewer prior to the interview.

Although participants were not explicitly told that the interviewer (AH) was a physiotherapist this was not concealed and in the majority of interviews it became evident to them, allowing a deep discussion about the physiotherapy interventions. Participants were aware that the interview was designed to aid the development of a physiotherapy intervention for people with dementia. Interviews were conducted face-to-face (with the exception of one which was by telephone at the participant’s request) and lasted approximately 30 min, although participants were given the opportunity to split the interview into two (or more) separate sessions, everybody was happy to complete the interview in one session. They were audio-recorded and transcribed verbatim immediately following completion of the interview. Memos were taken throughout the data collection and analysis phase and were used to guide discussion with the other authors while also refining the sampling strategy.

NVivo 11 (QSR International) was used to organize and code the data and facilitated a process of thematic analysis to be undertaken as suggested by Braun and Clarke [22]. Analyst triangulation [23] was carried out in order to increase the internal validity of the findings and involved two of the researchers independently analyzing a selection of the coded data and comparing findings. Two of the authors (AH and LB) independently coded the transcripts and discussed and compared coding strategies in order to reach consensus about coding and development of themes. Any disagreements in coding strategies were resolved by discussion, but the majority of disagreements revolved around variations in terminology and were easily resolved.

Identification of potential themes occurred throughout the data collection stage and was supported by the use of memos. These preliminary themes were structured into a thematic map and were further refined throughout the data analysis. The trustworthiness of the emerging themes was improved by discussion amongst aut-hors, using a process of peer debriefing [24] and involved the authors discussing the development of thematic maps in order to reach consensus on the emerging themes.

## Results

Three major themes and a further eight subthemes were formulated. The first theme “*physiotherapy is more than just a sheet of exercises”* explores the importance people with dementia and their carers placed on physiotherapists not just providing exercises but on providing support to enable the exercises to be undertaken successfully. Many of the participants described how resource pressures were perceived to affect the amount and type of physiotherapy they received and this forms the second theme. The final theme “the physiotherapy just vanished” explores the importance of the person with dementia and their carer having a shared understanding with the physiotherapist about the aims and goals for physiotherapy; in some cases a lack of this understanding resulted in a feeling of abandonment by the physiotherapist. The principles of PCC, such as acknowledging personhood, personalizing the person’s care, offering shared decision-making and prioritising the relationship as much as the interventions [25] were not experienced by our participants.

### Physiotherapy is more than just a sheet of exercises

Our participants described several factors that were important considerations for the physiotherapist to be able to help engage them and enable them to undertake appropriate exercises, going beyond simply handing out a set of exercises.

#### Understanding

The need for the physiotherapist to get to know the person was discussed by many participants, alongside an appreciation and understanding that people with dementia could still successfully be rehabilitated following injury or illness. Taking time to get to know the person was felt to be vital to allow the physiotherapist to develop individually tailored strategies to maximise engagement in their rehabilitation but it was commonly felt that physiotherapists failed to look beyond the dementia. Reasons for this were reported to be a lack of understanding about dementia but also a lack of time and resources available.

*“I think a lot of the problem lies that people look at someone with dementia and that’s all they see. They see someone with dementia. I mean I don’t look at my mum and see someone with dementia you know I see her as funny, witty, entertaining that is my mother. ” (carer – PA11 )*

Some participants felt that their physiotherapist lacked knowledge about treating older people and those with dementia and wanted to be recognised as needing an approach to treatment different from that received by younger people or those without dementia.

*“Yeah I think you’ve got to [inform the physiotherapist] because people understand better then otherwise they treat you like you’re an idiot if you’re not careful and as soon as people know then they are a little bit more…… they’ve got a little bit more respect in how they treat you.” (person with dementia – PA6)*

However, other participants reported feeling as though the “stigma” of having a diagnosis of dementia could negatively affect their care and wanted to receive the same treatment as somebody without dementia. This fear of receiving less satisfactory care, or being treated differently, if they disclosed the diagnosis of dementia led to several of our participants withholding their dementia diagnosis from their physiotherapist.

*“I didn’t tell him I had Alzheimer’s because you know as soon as you say you have Alzheimer’s ……. people instinctively think that you’re past it” (person with dementia, PA7)*

There was a fear that the physiotherapist may not understand how to treat them appropriately if the diagnosis was disclosed. There was also a reported embarrassment at having to disclose this information where the physiotherapist was not aware.

“*…..this young male physio that I go to wouldn’t understand a word if I said I had Alzheimer’s. So I haven’t said it to him, so it’s partially my fault but ……..I suppose it’s a question of self-respect.” (person with dementia, PA7)*

#### Giving confidence

The difficulty disclosing a diagnosis of dementia was frequently put down to a lack of confidence to share such information. It was reported by several participants that being given a diagnosis of dementia had significantly affected their confidence, both in terms of their health and in terms of managing everyday activities.

*“But the thing I’ve found now with Alzheimer’s is the lack of confidence of what you did used to do.” (person with dementia, PA10)*

Participants described this lack of confidence affecting their ability to engage with the physiotherapist and therefore felt unable to tell them that exercises were too complicated. They also lacked confidence to ask their physiotherapist to check that they were undertaking them correctly. Our participants reported trying to overcome this lack of confidence by having somebody else, usually their spouse, present during physiotherapy sessions.

Our participants reported feeling that the physiotherapist needed to appreciate that their lack of confidence might affect their ability to engage and adhere to the physiotherapy. They also felt that the role of the physiotherapist was to help develop a sense of improved confidence, which would allow them to undertake the physiotherapy correctly.

### Adapting treatment

The difficulties people with dementia and their carers reported with undertaking the physiotherapy meant that it needed to be adapted to make it possible for them to engage. However, different participants described varying adaptations that would help their ability to undertake the physiotherapy, highlighting the importance of individualizing treatment based on the exact needs of each person. None of our participants reported having an open discussion with their physiotherapist about how best to adapt their treatment to overcome any difficulties they experienced due to their dementia.

In practical terms, participants talked about treatments needing to be short and regular in order to create a routine to help them remember the physiotherapy.

*“So I think it’s just perseverance really and I think also yes making it a habit rather than a memory.” (person with dementia, PA11)*

The use of written exercise sheets was talked about favourably but these were not always offered even when requested. They gave the person with dementia confidence to be able to repeat the exercises correctly but in some cases were too complicated to follow.

#### Getting the right people involved

Involving the correct people in the treatment was reported to be something that was important but often poorly considered by the physiotherapist. Having a consistent physiotherapist providing input was reported by our participants to be invaluable but this was frequently impossible in acute settings.

*“I mean he [relative] used to complain a lot at the hospital ……… always changing the physio that’s the other thing you know about different people coming……. he didn’t like it when it was all different people all the time” (carer, PA9)*

Relatives were keen for others to be involved in the physiotherapy, such as day-care services or paid carers, but this was infrequently supported by the physiotherapists.

*“I was surprised they didn’t train the day care centre staff …….They do exercises with their clients in there anyway…..” (carer, PA4)*

The carers reported being happy to be involved in the physiotherapy if they were able, reporting that this was just “*part of their job”*. If they were not physically able to be involved in undertaking exercises, they wished to be involved in discussions and decision making. However, several carers found it difficult to assist with the exercises, or be involved in decision making, as their levels of carer burden were already too high.

“I said look this hasn’t broken my [relative] but I can tell you it has nearly broken me.” (carer, PA11)

### Lack of resources affected the physiotherapy

Resource limitations and pressures within the National Health Service in the UK (NHS) were frequently cited and our participants felt that these often negatively affected their care.

#### Difficulty accessing physiotherapy

Many of our participants described a difficulty accessing physiotherapy and when it was offered, it often took a long time to begin, which was perceived to negatively affect the ability to improve.

*“Well I think maybe you know by the time the physios got involved my [relative] has been lying in bed for a month.” (carer, PA11)*

In order to resolve this difficulty, several participants sought private physiotherapy input until the health services physiotherapy started. However, this then excluded them from receiving NHS physiotherapy.

*“once the NHS found out that he had private physio that was it they said they don’t want to do anymore……he is able to pay for someone himself.” (carer, PA9)*

There was also a reported difference in what could be offered from a private physiotherapist to what was offered by the NHS. Physiotherapists working for the NHS appeared more cautious with regards to health and safety, whereas the private physiotherapists were more flexible in their approach.

*“they [NHS physiotherapists] were wrapped up in health and safety you know when someone else [private physiotherapist] was managing to get him walking with just one of them they insisted on having two ……….the house was absolutely overcrowded with equipment because you know they would insist that he need a rotunda machine to stand and then another machine and then this sort of wheelchair …….. it ended up that there was hardly anywhere for him to walk in his house because of all this equipment.” (carer, PA9)*

#### Not getting what they deserved

Generally our participants had very low expectations of what physiotherapy they would receive and these low expectations were realized by many. These low expectations were grounded in an appreciation of a lack of resources available in healthcare as well as some previous negative experiences of physiotherapy interventions.

*“I suppose finances are difficult in there and the amount of people that need the service I’d say but that doesn’t sort of qualify her not having a service she’s entitled to.” (carer – PA3)*

However, while some participants were openly disappointed and felt that they did not get what they deserved, the majority accepted what was given – seemingly as they were unaware of what they were entitled to.

*“But I don’t know how long a piece of string is I’m not sure I wouldn’t have a clue I suppose that’s another thing you know how along the line of expectations of how long we would have expected them to keep trying.”(carer, PA9)*

Dissatisfaction usually revolved around a low number of sessions, poor treatments, frequent cancellations, or poor communication. When physiotherapy was started, it was often difficult to get ongoing physiotherapy and it was felt that the physiotherapists were frequently too quick to try to discharge people.

*“I still feel she could do with some more physio but my concern about it was, was in fact that having got the physiotherapy people to come and see her they were soon very keen to get shot.” (carer, PA3)*

### “The physiotherapy seemed to vanish”

Difficulty getting physiotherapy initially, followed by a lack of ongoing input was reported in combination with a frequent lack of clarity about how and why the physiotherapy ceased.

#### Poor communication

There was a lack of communication reported between the person with dementia and carer (where involved) and the physiotherapist, leading to a sense of confusion and unclear expectations. There was a lack of clarity and understanding reported by participants about the process of physiotherapy, what it was going to involve, and when it was completed. The role of the physiotherapist was unclear to a lot of participants and the use of physiotherapy assistants alongside qualified staff was poorly, or not, explained. There was no clear understanding of whether the physiotherapist was going to review them again, or whether the assistant was going to complete the course of treatment.

*“She [physiotherapist] said I’m going to go away and draw up a schedule and I’ll come back with the auxiliary and we’ll go through what’s to be done but in the event the physio didn’t come back she just sent the auxiliary” (person with dementia, PA2)*

This lack of effective communication led to confusion about when the physiotherapy was completed, leading to a feeling of abandonment. Participants described how the physiotherapist had just stopped attending, but there were no clear reasons for this.

*“she had a little bit and then it all sort of dropped off” (carer, PA1)*

#### Unclear goals

The lack of goals and aims appeared to precipitate a feeling of the physiotherapist “*vanishing*”. None of the participants had set goals with the physiotherapist before or during treatment. This led to confusion about what the aims of the treatment were and therefore an unclear ending to the physiotherapy. Timescales were not discussed or used as part of goal setting, creating confusion about how much physiotherapy was expected to be delivered.

*“No, she didn’t say you know in three months’ time you will look like …… no there was nothing like that. No she just said I’ll get some exercises for you that will improve your general wellbeing. I don’t think there was anything that said you know you will be able to do a particular thing after six months or something was there?” (person with dementia, PA2)*

Participants described the mental challenge that having no goals to achieve created. Progress was frequently slow and this made it difficult to see any improvements they were making.

*“I think you need a few more goals as well........... you need the sort of praise for it but also perhaps you need a few more goals you know. You know “really within another two months if should be back to”…” (person with dementia, PA7)*

## Discussion

The aim of this study was to explore the experiences of people with dementia receiving physiotherapy as well as the experiences of carers who were involved, in order to determine whether the principles of PCC were being applied to their treatment. Our participants described how they perceived resources to negatively affect the physiotherapy that was received, how physiotherapists needed to consider how to engage the person in physiotherapy rather than just providing exercises and also how a lack of understanding of the process of physiotherapy led to a feeling of desertion.

Participants experienced significant difficulty in obtaining physiotherapy and further difficulty receiving ongoing input. This was particularly evident in populations of people with dementia who fracture their hip, and a survey undertaken by the Chartered Society of Physiotherapy in conjunction with British Orthopaedic Association [26] suggested that less than half of people with dementia and hip fracture get referred for community based follow-up. A recent retrospective cohort study suggested similar figures, reporting 40.1% of people with dementia did not receive any physiotherapy following hip fracture [27]. The difficulty in obtaining physiotherapy initially and then receiving ongoing input led to several participants seeking private physiotherapy.

Patients reported difficulty undertaking the prescribed exercise which was viewed as poor adherence. However, participants described the lack of adaptation of treatments to meet their specific needs which made it impossible to adhere to treatments, a fundamental component that would be expected in PCC. Adherence to physiotherapy among people with dementia has not been explored but research in populations without cognitive difficulties suggests factors that may affect levels of adherence to physiotherapy interventions. Poor self-efficacy was suggested to limit adherence to physiotherapy in outpatient settings [28] and is commonly experienced by people with dementia due to a reduction in executive function and initiative. Adherence has also been identified as lower in people with high levels of depression [29], anxiety [30] and low self-motivation [31] which are all common problems faced by people with dementia. Our participants described the main assistance they needed was emotional support and understanding to try and overcome these difficulties. While carers were keen to be involved in the treatment generally, they required help to get their relative to undertake the physiotherapy as well as strategies to overcome behavioural difficulties that their relative may have demonstrated. Despite various factors affecting the choice of a patient adhering to treatment, it must be considered that failure to adapt the intervention to the individual needs of the patient may be the primary cause of lack of adherence.

Goal setting has been suggested to be an essential component of rehabilitation [32] and with people with dementia [33] despite there being a lack of high quality evidence supporting the effectiveness of goal setting in improving physical outcomes [34]. However, it has been suggested that goal setting may result in positive effects for psychosocial outcomes [34]. The lack of goal setting with our participants led to frustrations and confusion about the actual physiotherapy received and resulted in dissatisfaction with the input. It is suggested that goals of people with dementia may be less clear and well defined than for people where curing a disease is possible [33]. However, an appreciation that although the dementia cannot be ‘cured’, the condition they are receiving treatment for can still be ‘cured’ is vital for this population and goals should be carefully decided to consider this. However, it should be considered that although participants were unaware of goals being set for their treatment, this could potentially be related to a lack of understanding about what constituted a goal.

Lack of awareness of goals could be related to poor communication that was experienced between the physiotherapist and the patient. There was a common feeling that the physiotherapist was keen to discharge the person. This, in combination with a lack of understanding about what they were entitled to, often seems to have been the result of poor communication between those involved in their care resulting in unclear expectations. The feeling of desertion some of our participants felt is comparable to other research for patients who have needed long-term physiotherapy. The experience of people having had a stroke being discharged from physiotherapy has been reported similarly to often be one of “abandonment” [35] and may reflect an inability within the NHS to provide long-term ongoing care for people with long-term conditions.

The decision about whether to disclose a diagnosis of dementia has been explored in the literature [5]. People with ‘invisible conditions’ such as dementia may employ a strategy of preventative disclosure as described in literature relating to epilepsy [36]. Several of our participants withheld their diagnosis from their physiotherapist due to a concern that disclosing the diagnosis may negatively affect the care they received, while others felt it necessary in order for the physiotherapist to treat them effectively. While the perceived benefits of disclosing a diagnosis of dementia allowed compassion and understanding, the negative consequences of stigmatization were feared. This feeling of stigmatization or “social demotion” has previously been reported in people with a diagnosis of dementia and chronic illnesses [37] and affects the relationship between clinician and patient. However, the failure to disclose their diagnosis had the consequence of preventing the physiotherapist being able to adapt the treatment and personalise the intervention.

Dementia can be considered a medical ‘problem’ but is also a lived experience [5]. This is how our participants described it, with physiotherapy playing an important role in affecting this lived experience. It has previously been suggested that dementia’s historical biomedical background fails to appreciate the sociocultural aspects of the illness [5]. This is reflected in our study where participants wanted their physiotherapists to consider the greater context around which the exercises were prescribed but finding that these were often neglected in favour of a biomedical approach. Our participants described factors that were important for the physiotherapist to consider including using personal experiences of life and relationships, involving family and carers in decision making and building relationships between patient and healthcare professionals, all of which are essential components of PCC [38, 39]. However, none of our participants described their experience of physiotherapy as being one which employed a PCC approach to their treatment.

## Limitations

In order to increase the internal validity of the results, a process of analyst triangulation [23] was undertaken during the process of generating initial codes. It is suggested that having two (or more) researchers individually analyse the same data set, then compare their findings is suggested to reduce the potential researcher biases [23]. This process involved two researchers independently analysing a selection of the coded data and then findings were compared.

The primary researcher being a physiotherapist could be considered both a strength of the study as it allowed a deep discussion and shared understanding of what was received by the participants, or alternatively it could be considered a weakness as participants may have been less willing to portray a negative experience of physiotherapists. However, the interview questions were worded in such a way as to ensure that negative experiences could be openly discussed and it was made clear that hearing any negative experiences were important as well as hearing positive experiences.

Several interviews involved interviewing the carer and the relative together which added a challenging dimension to the interview, whereby the relative was often keen to answer for the person with dementia when they were struggling to think of an answer. However, during these interviews, the interviewer explained the importance of hearing the person with dementias’ views and they were asked to give them time to do so. Frequently the person with dementia sought reassurance from their relative when answering questions, perhaps further re-enforcing the lack of confidence that they felt.

The authors recognise the sample size was relatively small and the participants were recruited only from the South West of England, however the purposive sampling strategy aimed to recruit participants with a range of characteristics in order to increase the generalisability of the results. Furthermore, the results of this study are supported by a previous qualitative study we have undertaken whereby physiotherapists throughout the UK were sampled [17]. Physiotherapists reported appreciating the importance of incorporating PCC into their treatment of people with dementia, but were limited by resource pressures and lack of knowledge into using a PCC approach. The authors recognise that the results of this study may not be generalisable to other countries outside of the UK. The experiences reported were common amongst participants suggesting the sample size was sufficient to gain a good understanding of the experiences of this population while also being sufficient to answer the aims of the study.

## Conclusion

Our findings suggest that the principles of PCC were desired by participants, however, the incorporation of PCC principles into their individual management was rarely evident, with physiotherapists often approaching the patient from a biomedical perspective with little emphasis placed on the biopsychosocial aspects of their situation. Greater incorporation of PCC into the physiotherapy treatment of people with dementia may be very valuable in order to improve adherence to treatment.

Physiotherapists may need to develop other strategies to ensure that people with dementia get the input they need, such as improving the involvement of carers or incorporating exercises into more functional activities that can be undertaken with less supervision, while also promoting strategies that increase a person’s self-efficacy. We found that communication was often reported to be poor, particularly in relation to goal-setting and setting expectations of what physiotherapy could offer. A shared understanding of the role and aims of physiotherapy may help to avoid the frustration and feelings of dissatisfaction that our participants experienced.

## Additional file

Additional file 1: Interview topic guide. This document is the topic guide used by the researcher to structure all participant interviews. (DOCX 17 kb)

## Abbreviations

JDR: Join Dementia Research website
NHS: National Health Service, UK
PCC: Person centred care
