# Supplementary material for: Are physiotherapists employing person-centred care for people with dementia? An exploratory qualitative study examining the experiences of people with dementia and their carers
Source: BMC Geriatr. 2018 Mar 2;18:63. doi: 10.1186/s12877-018-0756-9 (PMC5834867; doi:10.1186/s12877-018-0756-9)
Supplement: Supplementary file 1 — Interview topic guide. This document is the topic guide used by the researcher to structure all participant interviews. (DOCX 17 kb) [file 12877_2018_756_MOESM1_ESM.docx]

**INTERVIEW QUESTIONS – JOINT INTERVIEW WITH DYADS**

Physiotherapy for people with dementia - experiences of patients and carers

**[Version 1.1]**

**1, Determine experiences of participants**

"Could you tell me about physiotherapy you have had in the past”

Explore;

- purposes
- setting receiving physiotherapy
- positive/negative
- outcomes
- carer’s perception of the physiotherapy

**2, Techniques used**

“Can you tell me about how the physiotherapist helped you do the exercises?”

Explore;

- method of delivery
- adaptation to account for cognitive problems
- cognition affect the outcomes?

**3, Involvement of carers**

“What involvement did you have with [patients name] doing their exercises?”

Explore;

- advice about how to help
- easy to help loved one?
- More effective if outsiders assisted
- others available to help? Eg support workers

**4, How could treatments be improved?**

“Could anything have been done differently to make the physiotherapy better for you both?”

Explore;

- Frequency
- Location
- Timings
- Different format ie. Video/paper based
- Group exercises?
- Adherence?
  - Until ‘better’? Got bored? Why did they stop? When did they stop? etc

**INTERVIEW QUESTIONS – PEOPLE WITH DEMENTIA**

Physiotherapy for people with dementia - experiences of patients and carers

**[Version 1.1]**

**1, Determine experiences of participants**

"Could you tell me about physiotherapy you have had in the past”

Explore;

- purposes
- setting receiving physiotherapy
- positive/negative
- outcomes

**2, Techniques used**

“Can you tell me about how the physiotherapist helped you do the exercises?”

Explore;

- method of delivery
- adaptation to account for cognitive problems
- cognition affect the outcomes?

**3, Involvement of carers/support workers**

“Did the physiotherapist ask anybody to help do the exercises with you?”

Explore;

- Carer involvement
- Happy to have carers involved
- Support workers

**4, How could treatments be improved?**

“Could anything have been done differently to make the physiotherapy better for you?”

Explore;

- Frequency
- Location
- Timings
- Different format ie. Video/paper based
- Group exercises?
- Adherence?
  - Until ‘better’? Got bored? Why did they stop? When did they stop? etc

**INTERVIEW QUESTIONS – NEXT OF KIN/CARERS**

Physiotherapy for people with dementia - experiences of patients and carers

**[Version 1.1]**

**1, Determine experiences of participants**

“Do you know why your relative/loved one required physiotherapy? Can you explain?”

**2, Techniques used**

“What did the physiotherapy involve?”

Explore;

- Adaptation for dementia
- Examples of intervention

**3, Carer involvement**

“Were you involved in their physiotherapy at all? What role did you play?”

Explore;

- referral
- Assessment
- Treatment
- Goal setting
- Support – emotional
- examples

**4, Could treatments be improved?**

“Could anything have been done differently to make the physiotherapy better for you or them?”

Explore;

- Timing
- Frequency
- Location
- Different methods?
- Continuity of physiotherapists
